# Positive effect of microvascular proliferation on functional recovery in experimental cervical spondylotic myelopathy

**DOI:** 10.3389/fnins.2024.1254600

**Published:** 2024-03-06

**Authors:** Xu-xiang Wang, Guang-sheng Li, Kang-heng Wang, Xiao-song Hu, Yong Hu

**Affiliations:** ^1^Department of Minimally Invasive Spine Surgery, The Affiliated Hospital of Guangdong Medical University, Zhanjiang, China; ^2^Department of Orthopaedics and Traumatology, The University of Hong Kong, Hong Kong, Hong Kong SAR, China; ^3^Orthopedics Center, The University of Hong Kong-Shenzhen Hospital, Shenzhen, China

**Keywords:** neural repair, microvascular proliferation, cervical spondylotic myelopathy, chronic, recovery

## Abstract

**Background and purpose:**

Cervical Spondylotic Myelopathy (CSM), the most common cause of spinal cord dysfunction globally, is a degenerative disease that results in non-violent, gradual, and long-lasting compression of the cervical spinal cord. The objective of this study was to investigate whether microvascular proliferation could positively affect neural function recovery in experimental cervical spondylotic myelopathy (CSM).

**Methods:**

A total of 60 male adult Sprague–Dawley (SD) were randomly divided into four groups: Control (CON), Compression (COM), Angiostasis (AS), and Angiogenesis (A G),with 15 rats in each group. Rats in the AS group received SU5416 to inhibit angiogenesis, while rats in the AG group received Deferoxamine (DFO) to promote angiogenesis. Motor and sensory functions were assessed using the Basso Beattie Bresnahan (BBB) scale and somatosensory evoked potential (SEP) examination. Neuropathological degeneration was evaluated by the number of neurons, Nissl bodies (NB), and the de-myelination of white matter detected by Hematoxylin & Eosin(HE), Toluidine Blue (TB), and Luxol Fast Blue (LFB) staining. Immunohistochemical (IHC) staining was used to observe the Neurovascular Unit (NVU).

**Results:**

Rats in the CON group exhibited normal locomotor function with full BBB score, normal SEP latency and amplitude. Among the other three groups, the AG group had the highest BBB score and the shortest SEP latency, while the AS group had the lowest BBB score and the most prolonged SEP latency. The SEP amplitude showed an opposite performance to the latency. Compared to the COM and AS groups, the AG group demonstrated significant neuronal restoration in gray matter and axonal remyelination in white matter. DFO promoted microvascular proliferation, especially in gray matter, and improved the survival of neuroglial cells. In contrast, SU-5416 inhibited the viability of neuroglial cells by reducing micro vessels.

**Conclusion:**

The microvascular status was closely related to NVU remodeling an-d functional recovery. Therefore, proliferation of micro vessels contributed to function -al recovery in experimental CSM, which may be associated with NVU remodeling.

## 1 Introduction

Cervical Spondylotic Myelopathy (CSM), the most common cause of spinal cord dysfunction globally, is a degenerative disease that results in non-violent, gradual, and long-lasting compression of the cervical spinal cord (Toledano and Bartleson, 2013). Histopathological studies have reported ischemic injury in the gray and white matter of the spinal cord in CSM. A cadaver study has observed impaired blood flow to axonal pathways (Breig et al., 1966). An experimental study by Karadimas et al. found loss and dysfunction of endothelial cells, resulting in subsequent vascular damage in a CSM model (Karadimas et al., 2013). Chronic and progressive compression of the cervical spinal cord results in microvascular damage and disruption of the blood-spinal cord barrier (BSCB) (Karadimas et al., 2013, 2015). Additionally, an animal study indicated that during the early stage of chronic compression, local pressure could reduce blood flow, which is potentially related to vascular occlusion following compression (Kurokawa et al., 2011; Huang et al., 2022a). Recently, Yu et al. (2021) provided evidence that chronic spinal cord compression leads to vascular redistribution and neovascularization, with the latter primarily occurring in the anterior horn of the compressed area in the spinal cord tissue. A previous study suggested that neurological function improvement occurs concomitantly with microvascular proliferation (Li et al., 2023). Another study reported that Neurovascular Unit (NVU) compensation might contribute to functional recovery after CSM (Schaeffer and Iadecola, 2021; Li et al., 2023). A previous study suggested that endothelial cell damage and BSCB disruption cause neurological dysfunction (Li et al., 2022). However, it remains unclear whether microvascular proliferation plays a role in alleviating neural damage progression in experimental CSM. Therefore, we hypothesize that microvascular proliferation may contribute to spinal neural repair processes in the CSM model. To verify this hypothesis, we established a CSM model, altered the NVU status, and compared spinal cord motor and sensory functions and pathological manifestations.

## 2 Materials and methods

### 2.1 Compression material and drugs

A water-absorbing and progressive expandable synthetic polyurethane polymer sheet was used as an implanting compression material. The polyurethane polymer sheet was made of isocyanates and polyols (Guangzhou Fischer Chemical Co., Ltd., Guangzhou, China). A polymer sheet at the size of 1 mm × 1.5 mm × 4.0 mm was cut and sterilized for implantation preparation. SU5416 (GLPBIO; Catalog No.: GC15307; Cas No.: 204005–46-9), 3-(3,5-dimethyl-1H-pyrrol-2-ylmethylene)-1,3-dihydroindol-2-one, is a selective potent inhibitor of VEGFR-2 tyrosine kinase (Fong et al., 1999; Mendel et al., 2000). Referring to the effect of SU5416 in inhibiting angiogenesis in relevant literature and preliminary experiments, in formal experiments, each rat (AS group) was injected with (i.p.) the drug (15 mg/kg, SU5416 was dissolved in dimethylsulphoxide, DMSO) once a day before surgery until the day of tissues acquisition (Skold et al., 2006). Deferoxamine (DFO, 100 mg/kg per day, Novartis, Basel, Switzerland, 500 mg dissolved in 5 mL of 0.9% normal saline) upregulated the HIF-1α/VEGF signaling pathway to promote angiogenesis (Yao et al., 2019). The drug was injected half an hour before compression surgery and once a day after surgery until the rat spinal cord tissues were obtained. Rats in CON and COM groups were administered an equal amount of DMSO and saline each day. Rats in the AS group were administered an equal volume of saline, and rats in the AG group were administered an equal volume of DMSO.

### 2.2 Grouping and modeling

A total of 60 male adult Sprague–Dawley (SD) rats, weighing between 250 and 300 grams, were divided into four groups: Control (CON), Compression (COM), Angiostasis (AS), and Angiogenesis (AG), with 15 rats in each group. The model period lasted 4 weeks. After skin preparation, an incision was made along the midline posterior neck of the rats, approximately 3 cm in length. A muscular indentation was observed at the craniocervical junction region. Forceps were used to lift the muscle and create a cavity, followed by a transverse incision along the midline. A muscular enlargement was visible around the C2 ~ C3 extending toward the paravertebral muscle. Upon separating the paravertebral muscle, the C4 lamina was identified. The front end of the curette was gently inserted into the C4/C5 interlaminar space to open the left lamina. The ligamentum flavum and a portion of the lamina were removed to access the epidural space. A polyurethane membrane (1 mm × 1.5 mm × 4.0 mm) was implanted into the epidural space at the C5/C6 level, designed to absorb water and expand, exerting continuous, progressive compression on the spinal cord (Li et al., 2022). Care was taken to avoid spinal cord injury throughout the procedure. Rats in the CON group only had their vertebral plates opened, without the insertion of compressive materials. Antibiotics were administered intraperitoneally daily for 3 days following the surgery.

### 2.3 Neurological function assessment

Locomotor function was evaluated using a 21-point BBB scale system in an open field (Basso et al., 1996). The time points of the BBB scale assessments were set at days 1, 3, 7, 14, 21, and 28 after the operation. Two spinal surgeons independently evaluated the locomotor function of the rats in the study. The average score was calculated to illustrate the dynamic change in motor function. Measurements of SEP were performed on rats after administering general anesthesia (40 mg/kg, 1 mL/kg). Sensory function integrity was evaluated using SEP with our established protocol. We stimulated the median nerve in rats using electrode needles. The stimulus parameters were generally set to a square wave of 5.1 Hz and an amplitude of 0.1 ms. An evoked potential recording system (YRKJ-A2004, Zhuhai Yirui Technology Co., Ltd., China) was used to record SEP signals. The signal obtained from each measurement was superimposed 500 times, resulting in a better final SEP quality.

### 2.4 *Lycopersicon Esculentum* agglutinin staining

The rat tails were fixed and wiped repeatedly with 75% alcohol until the tail veins were clearly visible. *Lycopersicon Esculentum* Agglutinin (LEA, L0401, FITC conjugate, Sigma-Aldrich) was then injected into the tail veins (Robertson et al., 2015; Zhan et al., 2020), followed by a 30-min waiting period. LEA could bind strongly to poly-N-acetyllactosamine oligosaccharides, which are the main components of carbohydrate substances on the surface of vascular endothelial cells (Pashkunova-Martic et al., 2011). Subsequently, observations were conducted using a confocal microscope. Rats were euthanized with an overdose of pentobarbital sodium. After compressing the tissue, the corresponding segment of the cervical cord was rapidly acquired. A cryomicrotome was used to cut the cervical spinal cords into 3 μm frozen sections. The fluorescence wavelength was set at 540 nm.

### 2.5 Tissues preparation and histopathological examination

All rats were perfused with heparin-saline solution (50 ml) through the ascending aorta, followed by paraformaldehyde/glutaraldehyde mixed solution (300 ml, 4%/2.5%, DF0149, Beijing Leagene Biotechnology Co., Ltd). After perfusion, the cervical spinal cords of the corresponding segments were placed in 4% paraformaldehyde for fixation for 10–15 h. The tissues were then dehydrated, cleared, and embedded in paraffin. The tissues were sectioned into paraffin slices and subjected to deparaffinization, rehydration, and various pathological staining procedures. With HE staining solution (G1120, Solarbio Life Science, China), the cell nuclei were stained blue, while the cytoplasm and extracellular matrix were stained red. The TB staining solution (Toluidine Blue Method, G1436, Solarbio Life Science, China) is a basic dye that identifies the basophilic NB in motor neurons. Myelin cells and cell membranes enveloping axons can be stained with the LFB (G3242, Solarbio Life Science, China) staining solution, resulting in a blue color against a colorless or light blue background. After pathological staining, the samples were observed under a light microscope (FV-1000, Olympus, Japan). The captured pictures were then analyzed using Image J software.

### 2.6 IHC staining

Paraffin sections were uniformly dewaxed and rehydrated. The antigen retrieval solution (C1034, Solarbio Life Science, China) was diluted at a ratio of 1:50. The solution, along with the paraffin section, was placed into the microwave oven, heated for 15 min, and then allowed to cool to room temperature naturally. To enhance the antigen–antibody reaction, the target antigen in the cytoplasm or nucleus needed to be exposed to the cell membrane using Triton X-100 (1,1,000, ST795, Beyotime Biotech. Inc.). This step was performed to increase antigen availability for antibody binding. An appropriate amount of 3% H2O2 (SA1020, Boster Biological Technology Co., Ltd.) liquid was added to each paraffin section to completely cover the tissue. This treatment was left for 15 min at room temperature to reduce non-specific staining. A 5% concentration of Bovine Serum Albumin (5% BSA) was applied onto the tissue and sealed at 37C for 1 h. The tissue samples were then incubated with CD31 (Mouse Monoclonal Antibody, Abcam, ab64543; 1:500), NEUN (Rabbit Monoclonal Antibody, Abcam, ab177487; 1:500), GFAP (Rabbit polyclonal Antibody, Abcam, ab7260; 1:1500), Olig2 (Rabbit monoclonal Antibody, Abcam, ab109186; 1:500), and IBA (Rabbit monoclonal Antibody, Abcam, ab178846; 1:500). After dropwise addition of these antibodies, the tissue samples were incubated in a 4°C freezer for 10 ~ 15 h. The diluted second antibody (Goat anti-rabbit IgG H&L-HRP, Abcam, ab6721, 1:500; Goats anti-mouse IgG H&L-HRP, Abcam, ab6789, 1:500) was added to cover the sample and incubated at 37°C for 1 h. The DAB solution (Item No.: AR1022, Boster Biological Technology Co., Ltd) was added, and staining was stopped based on real-time results. Finally, the process of dehydration, clearing, encapsulation of neutral resin, and optimal picture acquisition under the light microscope was performed.

### 2.7 Statistical analysis

SPSS 24.0 software (SPSS, Inc., Chicago, IL, United States) was used for statistical analysis, and GraphPad Prism 9.4.0 software (GraphPad Software Inc., San Diego, CA, United States) was used for graphing. The measured data were expressed as mean ± SEM. R software (version 4.1.0) was used to create heatmaps. The data obtained through various measurement methods were analyzed using one-way ANOVA. The significance level was set at α = 0.05. A *p*-value less than 0.05 was considered statistically significant, indicating a significant difference between groups.

## 3 Results

### 3.1 Functional recovery promotion after DFO intervention

**Figure 1:**
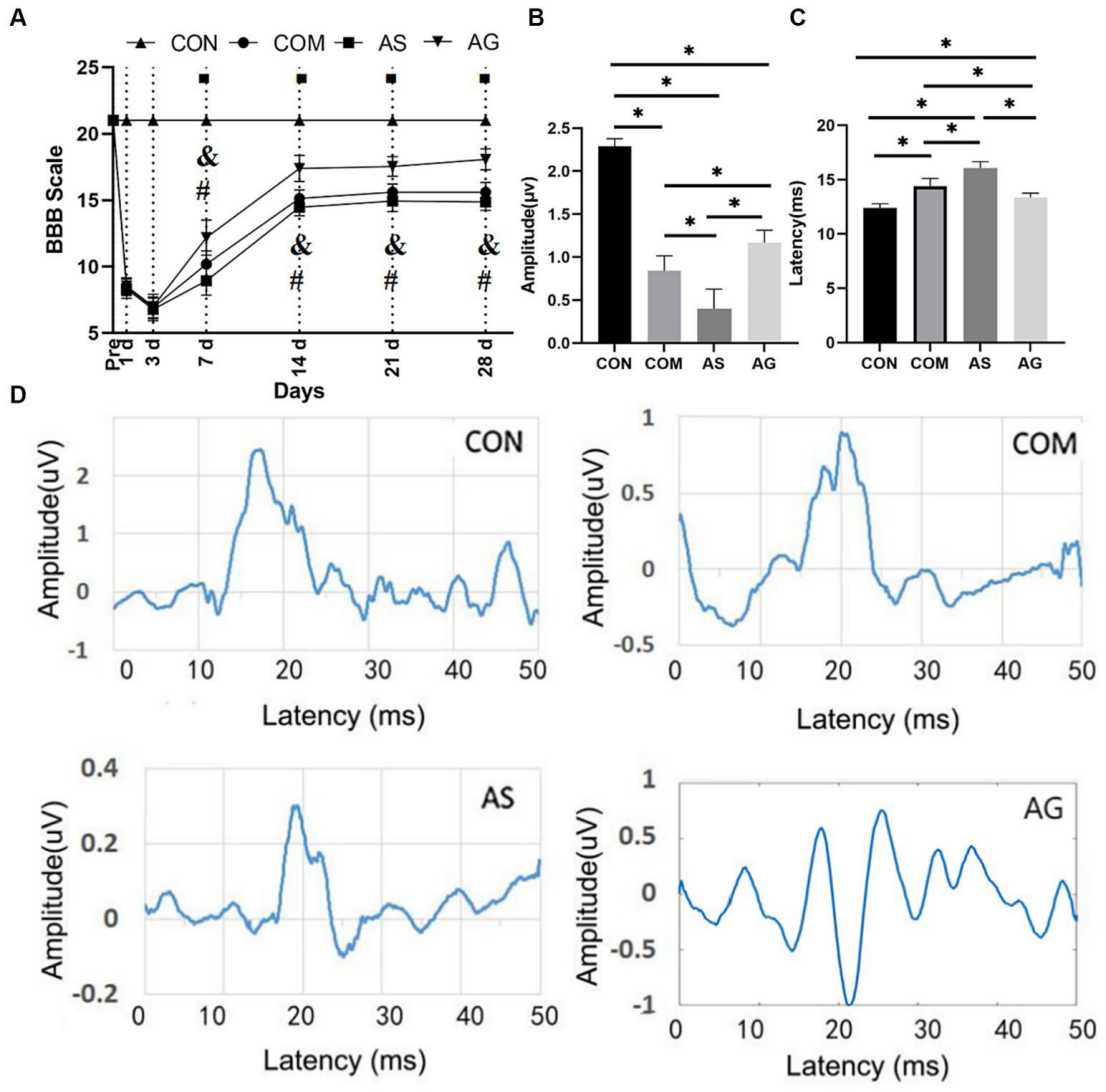
Functional recovery promotion after DFO intervention. **(A)** On days 7, 14, 21, and 28, there were significant differences in the BBB score among the COM, AS, and AG groups (*p < 0.05*), and the AG group had the highest score among the experimental group. **(C)** The AG group had the shortest SEP latency among the experimental groups. **(B)** The amplitude demonstrated an opposite performance to the latency. The comparison between any two of the three groups showed significant differences (*p < 0.05*). **(D)** A representative SEP waveform in each group. In **A**, “◾” indicates a statistical significance in the differences between the COM, AS, and AG groups when compared to the CON group (*p < 0.05*); “&” signals a statistical significance in the differences between the CON, AS, and AG groups when compared to the COM group (*p < 0.05*) and “#” signifies a statistically significant difference between the AS and AG groups (*p < 0.05*); in **B,C**, “*” signifies that there was statistical significance (*p < 0.05*).

The CON group demonstrated normal locomotor function, consistently scoring a BBB of 21. In the other three groups, i.e., AG, COM, and AS groups, BBB scores were lower than those in the CON group. In experimental CSM, there were no significant differences in the BBB scores among the three groups on days 1 and 3. From day 7 onwards, the AG group consistently had the highest BBB scores, followed by the COM group, with the AS group having the lowest BBB scores (Figure 1A; *p < 0.05*). The latency of SEP in the COM group remained more prolonged than that of the CON group (Figure 1C; *p < 0.05*). In the AG group, which received a DFO intervention, the SEP latency was significantly shorter (Figure 1C; *p < 0.05*). Among the four groups, the AS group showed the most prolonged latency, which was significantly different from the others (Figure 1C; *p < 0.05*). In comparison with the SEP amplitude, the CON group had the highest amplitude, followed by the AG group and the COM group (Figure 1B), while the AS group had the lowest amplitude (Figure 1B; *p < 0.05*).

### 3.2 Neuronal restoration after DFO intervention

**Figure 2:**
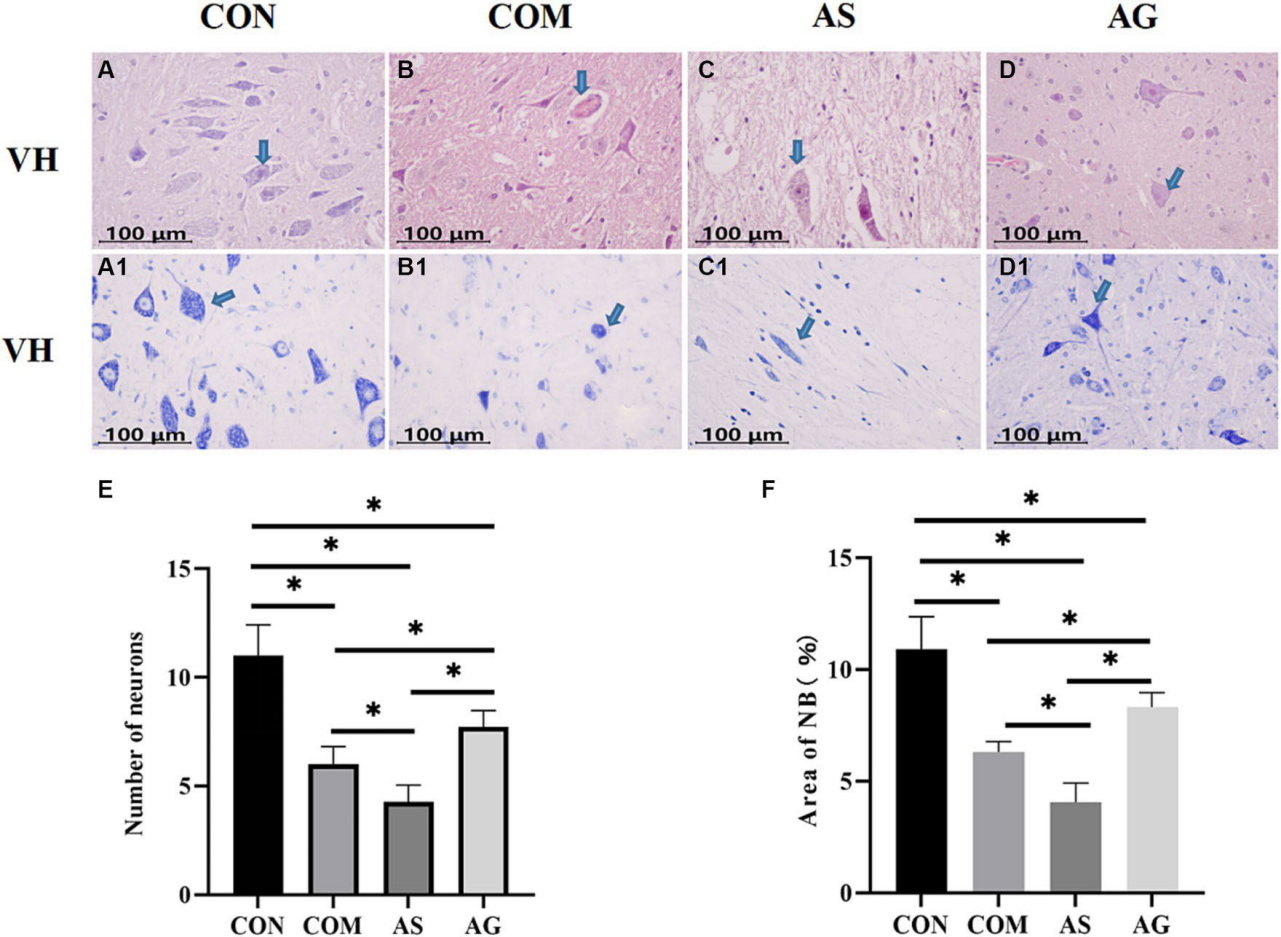
Neuronal restoration after DFO intervention. **(A–E)** A significant decline in the number of polygonal neurons was discovered in the COM group, exacerbated in the AS group, and restored in the AG group. **(A1, B1, C1, D1, F)** The changes in NB content mirrored the trend of neuronal quantity (indicated by an arrow). A significant reduction in the NB content was observed in the COM group, which further diminished in the AS group but rose in the AG group, suggesting a positive effect of DFO and a negative effect of SU5416 on neural restoration. VH, ventral horn; NB, Nissl bodies. “*” signifies statistical significance (*p < 0.05*).

In the CON group, there was a significantly high number of polygonal motor neurons characterized by a substantial presence of Nissl bodies (NB) (Figures 2A and A1). As observed by HE staining, the neuronal number significantly reduced in the gray matter after spinal cord compression (Figures 2A,B; *p < 0.05*). A significant reduction in the number of neurons was observed in the AS group (Figures 2B,C; *p < 0.05*). Compared to the COM and AS groups, the number of polygonal motor neurons in the gray matter was significantly increased in the AG group (Figures 2B,D; *p < 0.05*). Abundant NBs, with profound staining intensity, were observed within the neuronal cell bodies and dendrites of the CON group (Figure 2A1). In the AG group, the content of NB significantly increased (Figures 2B1 and D1; *p < 0.05*), while it decreased in the AS group (Figures 2B1 and D1; *p < 0.05*), compared to the COM group.

### 3.3 Axonal remyelination in white matter after DFO intervention

**Figure 3:**
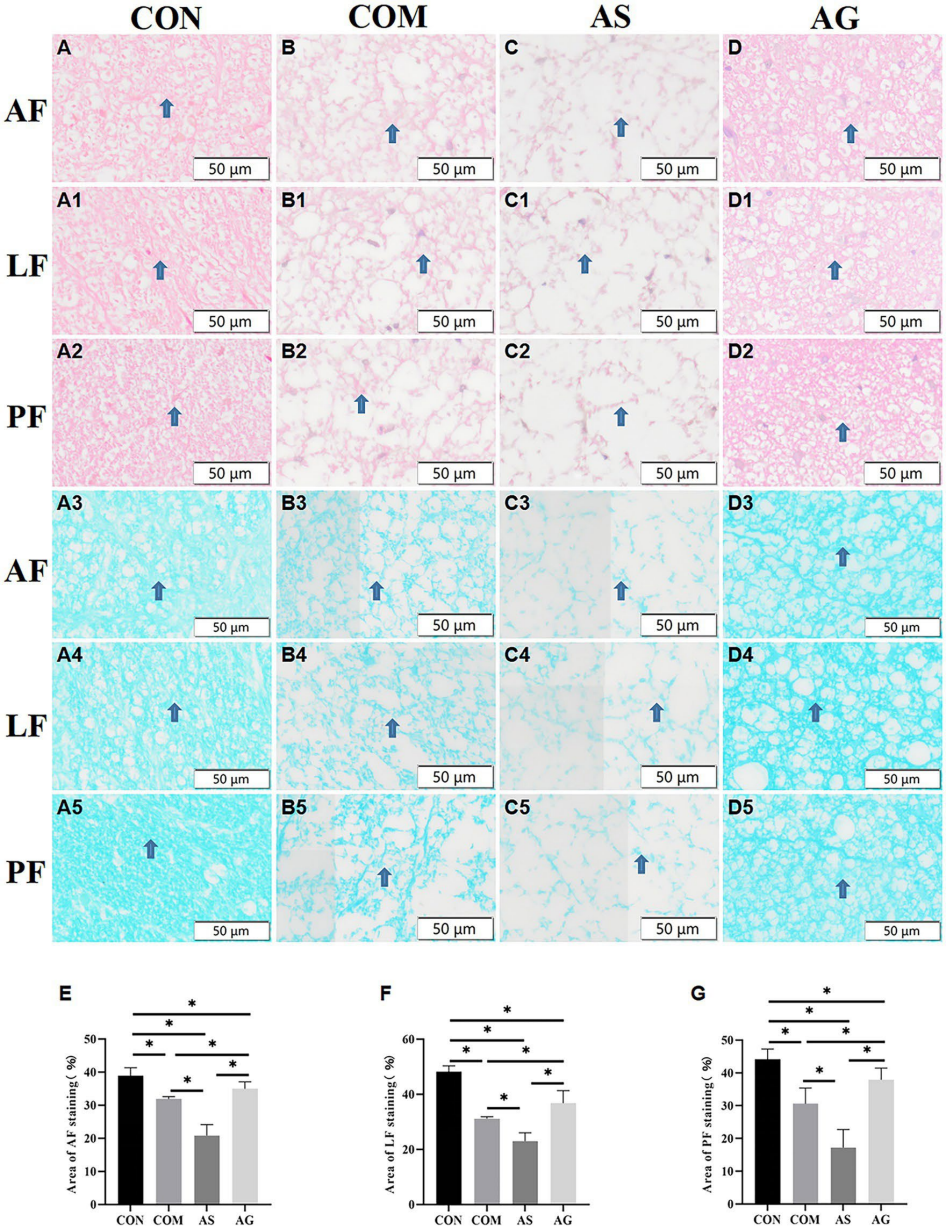
Axonal remyelination in white matter after DFO intervention. **(A3–A5, B3-B5)** After compression, the myelin sheath of the AF, LF, and PF sections of the spinal cord was stained light blue (as indicated by arrows), displaying partial axonal rupture. **(A–A5, B3-B5, C-C5, D-D5)** Vacuolization was observed in the AF, LF, and PF sections of the spinal cord. **(B-B5, C-C5, D-D5, E, F, G)** The COM group displayed a decrease in myelin sheath and axonal integrity in the AF, LF, and PF, while these pathological features were more pronounced in the AS group but showed partial improvement in the AG group (*p < 0.05*). AF, Anterior funiculus; LF, Lateral funiculus; PF, Posterior funiculus; “*” signifies that there was statistical significance (*p < 0.05*).

The myelinated axons of the neural fiber in the white matter of the Anterior funiculus (AF), Lateral funiculus (LF), Posterior funiculus (PF) areas showed organized patterns and intense staining when assessed with the HE staining (Figures 3A, A1, and A2). Evident disruption of neural fibers, vacuolated change, and axon-myelin separations were seen in all areas of the COM group (Figures 3B, B1, and B2). The AS group exhibited more severe axonal disruptions or ruptures than those seen in the CON group (Figures 3C, C1, and C2; *p < 0.05*), while the AG group showed significant improvement compared with the COM and AS groups (Figures 3D, D1, and D2; *p < 0.05*).

The myelin sheaths of AF, LF, and PF were stained with various degrees of blue. The interrupted myelin edges appeared curled, and multiple unstained vacuoles were present between the neural fibers. The different areas of white matter exhibited organized structure and blue-staining in the CON group (Figures 3A3, A4, and A5). Decolorization of blue and remarkable vacuolation can be identified in white matter in the COM group (Figures 3B3, B4, and B5), especially in the AS group (Figures 3C3, C4, and C5), and the myelin areas of the two groups decreased significantly compared to that of the CON group (Figures 3E–G; including AF, LF, and PF; *p < 0.05*). The AS group had the smallest staining area (Figures 3E–G). On the contrary, the myelin areas increased significantly in the AG group compared to the COM and AS groups (Figures 3D3-D5; E–G; and *p < 0.05*), suggesting remyelination of axons in white matter.

### 3.4 Microvascular proliferation and thereby NVU remodeling after DFO intervention

**Figure 4:**
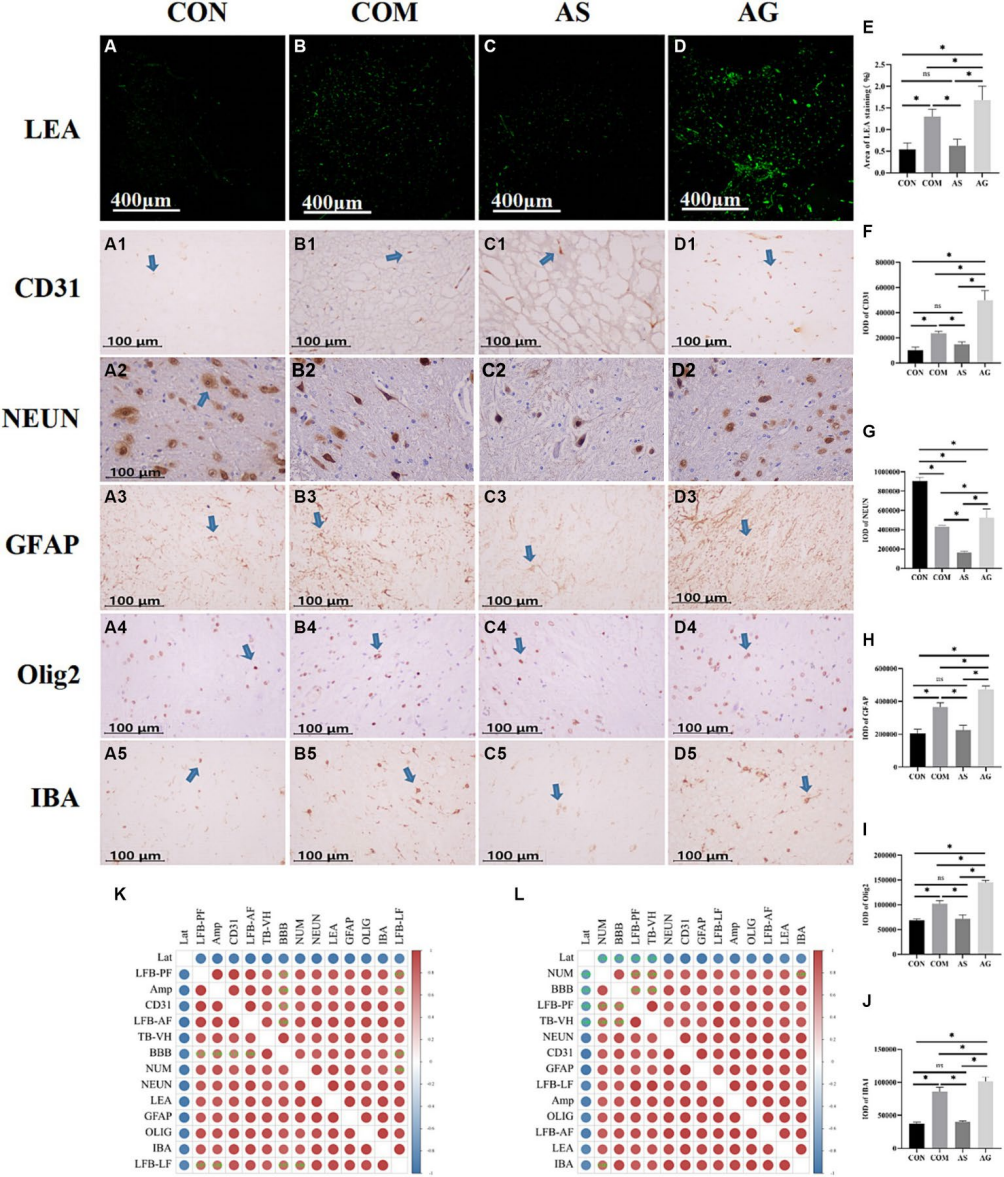
Microvascular proliferation and, thereby, NVU remodeling after DFO intervention. **(A–D, A1-D1, E, F)** Semiquantitative analysis of LEA staining and CD31 (indicated by an arrow) revealed that SU5416 intervention inhibited endothelial cell proliferation, while DFO intervention promoted it (indicated by an arrow). **(A2–D2, G)** Expression of NEUN was lower in the COM group than in the CON group and declined in the AS group. In the AG group, it increased. **(A3-D3, A4–D4, A5-D5, H, I, J)** Compared to the CON group, there was an increase in glial cells in the COM group. Glial cells decreased in response to SU5416 but increased with DFO. **(K, L)** Heatmap analysis showed that the components of NVU were closely related to motor and sensory functions, as well as micro-pathological changes in the spinal cords. Lat, Latency; Amp, Amplitude; TB, Toluidine Blue; LFB, Luxol Fast Blue; AF, Anterior funiculus; LF, Lateral funiculus; PF, Posterior funiculus; VH, ventral horn; BBB, Basso Beattie Bresnahan; NUM, number of neurons; LEA, *Lycopersicon Esculentum* Agglutinin; CD31, markers for microvessels; NEUN, markers for neurons; GFAP, markers for astrocytes; Olig2, markers for oligodendrocytes; IBA, markers for microglia; “*” signifies that there was statistical significance (*p < 0.05*).

Brownish-yellow or tan-specific NEUN staining was seen in the CON, COM, AS, and AG groups. The shape of neurons was outlined as polygonal, triangular, round, or oval. Some neurons had nuclei that were clearly visible and stained a dark brown color. Astrocytes showed small patches of staining with focal radial patterns. Oligodendrocytes appeared solid, hollow round, or circular-like after staining. Microglial staining was morphologically variable and mostly either round or patchy. Vascular marker levels in the CON group were stable. The microvessels showed a significant increase after compression (Figures 4A vs B, for LEA, Figure 4E; Figures 4A1 vs B1, for CD31, Figure 4F; *p < 0.05*). When compared to the COM group, SU5416 decreased vascular markers (Figures 4B vs C, E, B1 vs C1, F; *p < 0.05*), while DFO increased it (Figures 4B vs D, E, B1 vs D1, F; *p < 0.05*). The expression level of NEUN in the CON group remained consistently high but declined after prolonged compression (Figures 4A2 vs B2, G; *p < 0.05*). This condition was worsened after the addition of SU5416 (Figures 4B2 vs C2, G; *p < 0.05*) although it was effectively mitigated by DFO (Figures 4B2 vs D2, G; *p < 0.05*).

In the experimental CSM group, glial cells were found to have increased to varying degrees (Figures 4A3 vs B3, A4 vs B4, A5 vs B5, H–J; *p < 0.05*). The SU5416 intervention inhibited the expression of glial cells biomarkers (Figures 4B3 vs C3, B4 vs C4, B5 vs C5, H–J; *p < 0.05*), while DFO promoted survival (Figures 4B3 vs D3, B4 vs D4, B5 vs D5, H–J; *p < 0.05*). Glial cells underwent changes in response to alterations in vascular endothelial expression. We created a heatmap using R software. The bar on the right side represents the correlation. Red denotes a positive correlation and blue denotes a negative correlation. The darker the red or blue color, the stronger the correlation. The levels of expression of NEUN, GFAP, Olig2, IBA, CD31, and LEA were found to be significantly correlated in the heatmap after the interventions of SU5416 and DFO (Figures 4K,L; *p < 0.05*). The changes in motor and sensory functions, alterations in the number of neurons, variations in NB counts, and degree of white matter demyelination were all closely related to the NVU and had statistical significance (Figures 4K,L; *p < 0.05*).

## 4 Discussion

This study aimed to investigate whether microvascular proliferation could positively affect the recovery of spinal cord neural functions after CSM. In the preliminary experiment, the mortality rate of SD rats subjected to a severe CSM model increased. There was no significant short-term recovery in the motor function of the rats, which could potentially mask the role of microvascular proliferation in facilitating functional recovery. The severe CSM model also failed to quantitatively analyze the recovery of neural function using electrophysiological techniques, making it difficult to discover the correlation between microvascular proliferation and functional recovery. Therefore, in this study, rats received mild spinal cord compression to demonstrate the role of microvascular proliferation in spinal cord injury. After constructing the mild CSM model, rats had the potential for short-term recovery of neural functions, which in turn was conducive to the assessment of neurological functions. It was possible to verify the research objectives by promoting and inhibiting vascular proliferation. Changes in neural function and neurovascular structure after CSM were investigated in three different conditions, i.e., spontaneous, microvascular proliferation, and microvascular inhibition. To verify the scientific hypothesis, this study designed four groups with different degrees of microvascular proliferation for comparison. The CON group served as a control group to obtain normal data from animals with intact spinal cord function. The COM group served as a CSM model to observe natural progression after chronic compression to the cervical spinal cord, including neurofunctional degeneration and significant microvascular proliferation compared to the CON group. On the basis of the nature of the CSM group, the AS group was administered SU5416 with the purpose of inhibiting microvascular proliferation, while the AG group was administered DFO to promote microvascular proliferation. If promoting or inhibiting microvascular proliferation resulted in an increase or decrease in the relevant indicators in the CSM model, the scientific hypothesis would be validated. The macroscopic evaluation of neural function was performed using BBB and SEP techniques. The results of this study demonstrated the positive effect of microvascular proliferation on neural function recovery, while microvascular inhibition had a negative effect. The histology and immunohistochemical study further investigate the microstructures in the spinal cord of various microvascular statuses to verify the effect of microvascular proliferation on neurological changes. This study provides evidence to support the positive effect of microvascular proliferation on the recovery of spinal cord neural functions after CSM. It suggests a potential new strategy for the clinical treatment of CSM.

BBB reflects the coordination of joint movement and function well. The BBB scale of the COM, AS, and AG groups showed a significant decline compared to the CON group on postoperative days 1 and 3. As for the sign of successful modeling, it was primarily assessed to a BBB score in a range of 6.5 to 7 at three days post-modeling (Basso et al., 1996). In the first 3 days of BBB evaluation, the AG group did not show significant differences with the AS and COM groups. In the 28 days after CSM, the AG group showed the highest BBB score, indicating a significant improvement in recovery of motor function after CSM. On the contrary, the AS group showed the lowest BBB in the 28 days after CSM. The COM group showed a modest BBB compared to the AG and AS groups. Rats in the AG group were more active than those in the AS group. This finding is in agreement with previously reported results that angiogenesis promotion in injured spinal cords enhances functional recovery in experimental spinal cord injury studies (Kumar et al., 2018; Chen et al., 2022; Hong et al., 2022; Huang et al., 2022b). It was suggested that microvascular proliferation would improve motor function recovery, but microvascular inhibition would obstruct motor function recovery.

SEP technology records the electrical activity of the sensory neural pathway, reflecting the sensory function (Peeling et al., 2010; Muzyka and Estephan, 2019; Cui et al., 2021; Li et al., 2021). SEP is usually measured by latency and amplitude. The latency of evoked potentials is determined by the conduction speed of nerve fibers, while the amplitude is determined by the number of neurons and the synchronicity of their activity. In this study, the AG group showed a significantly higher amplitude and shorter latency in SEP measurement. At the other end, the AS group showed a significantly lower amplitude and longer latency, while the COM group was measured between the AG and AS groups. This presents a similar effect of microvascular status on sensory function recovery after CSM. Both BBB and SEP tests prove the positive effect of microvascular proliferation on the recovery of spinal cord neural functions after CSM. Alterations in the latency and amplitude of SEP can be explained by changes in the ultrastructure of neurons. The number of active blood vessels may be directly related to the oxygen supply to spinal cord tissue. The TB staining method is used for staining Nissl substances and neurons in paraffin-embedded tissue sections. The presence or absence of NB is an important indicator of nerve cell damage. Significant neuronal loss, cell swelling, pericellular space enlargement, and neuronal atrophy have been observed in a brain injury rat model (Hou et al., 2019). It has been noted that NB is very sensitive to hypoxia (Hou et al., 2019). Developing neurons require the attraction of blood vessels to provide nutrients for their growth (Martins et al., 2022; Zheng et al., 2023).

In terms of the loss of neuronal numbers, the reduction in NB content is consistent with this study. Hypoxia can also lead to demyelination in white matter. The LFB staining can demonstrate the integrity, degeneration, and necrosis of myelin sheaths. When nerve fibers are damaged, changes in myelin sheaths such as expansion, coiling into spherical shapes, fragmentation, or complete loss of sheaths can occur. Normal spinal cord myelin can be stained as a continuous and natural sky-blue color. Compared to the CON group, the AG group had the mildest degree of demyelination, the AS group had the most severe, and the COM group was in between. The death of oligodendrocytes was the main cause of demyelination of spinal cord axons (Mifsud et al., 2014). Oligodendrocytes were almost as sensitive to hypoxia and ischemia as neurons. Their vulnerability to hypoxia and ischemia was related to cell maturity. Experiments confirmed that advanced oligodendrocytes progenitor cells and immature oligodendrocytes died rapidly after ischemia initiation. Compared to the COM group, the AG group had a significant increase in oligodendrocytes (Figures 4D4 vs B4; *p* < 0.05), while the AS group had a significant decrease (Figures 4C4 vs B4; *p* < 0.05). IHC and LEA were used for semi-quantitative analysis of the NVU, CD31 as the vascular marker, NEUN as the neuronal marker, GFAP as the astrocyte marker, Olig2 as the oligodendrocyte marker, and IBA as the microglial marker. The changes in the expression levels of GFAP, Olig2, and IBA in each group were consistent with those in microvessels. NEUN expression was highest in the CON group. The alteration of NEUN expression in the other three groups was consistent with the changes in neuron number and the content of NB. Through a heatmap analysis, we found that the components of the NVU are closely interconnected and have a correlated relationship with neural function and structure. The alteration of the microvascular state may cause a series of changes in glial cells, ultimately promoting neural repair. Liddelow et al. (2017) believed that astrocytes can be divided into A1 and A2 types based on their phenotype. A1-type astrocytes are thought to be neurologically harmful (Kisucká et al., 2021). On the other hand, A2 astrocytes are believed to have neuroprotective effects that promote cell proliferation and survival while inhibiting apoptosis (Fujita et al., 2018). Microglia can also be divided into classically activated microglia/macrophages (M1 type, which promoted the maintenance of inflammation) and alternatively activated macrophages (M2, which inhibited inflammation) according to the activation mode. In the CNS, myelin formation is closely related to oligodendrocytes, which form a functional unit with axons and play a crucial role in axonal integrity. The death of oligodendrocytes was the main cause of demyelination of spinal cord axons (Mifsud et al., 2014). Compared to the COM group, the AG group had a significant increase in oligodendrocytes (Figures 4D4 vs B4; *p < 0.05*), while the AS group had a significant decrease (Figures 4C4 vs B4; *p < 0.05*). This is consistent with the role of oligodendrocytes in literature (Fern and Möller, 2000; Back et al., 2002).

In this study, as an important part of the NVU, microvessels underwent changes that resulted in positive alterations in neural repair. Endothelial cell damage and disruption of the blood-spinal cord barrier, inflammation, and apoptosis were the most common pathological manifestations of CSM. Therefore, interventions targeting the pathological changes of CSM, such as those in microvessels, might achieve significant effects. However, studies on direct microvascular interventions in the CSM model have been few. Currently, a few biological studies have focused on vascular reconstruction in spinal cord injury (SCI). Substances such as collagen, hyaluronic acid (HA), and fibronectin have been found to enhance angiogenesis and promote axonal regeneration after spinal cord injury (Wei et al., 2010; Wang et al., 2018; Yao et al., 2018; Loureiro et al., 2019). Following spinal cord injury, certain synthetic materials are capable of providing a sustained release of angiogenic factors to the lesion site (Patist et al., 2004; Ropper et al., 2017). Vascular endothelial growth factor (VEGF) can stimulate angiogenesis and axonal regeneration (Widenfalk et al., 2003). Estrogen and Chondroitinase ABC can also promote angiogenesis and may be effective for neurofunctional recovery after SCI (Milbreta et al., 2014; Ni et al., 2018). In TRPV4 knockout (KO) mice, we have observed reduced endothelial cell damage, increased expression of tight junction proteins, and improved neurofunction (Kumar et al., 2020).

### 4.1 Limitations

This study does face several limitations. First, the intervention dosage of DFO and SU5416 was based on references, which affirm that increased microvascular proliferation in the CSM model facilitates the recovery of spinal cord neural function. However, it remains unclear at which level of microvascular proliferation the maximum promotion of spinal cord neural repair occurs. It is also uncertain whether microvascular proliferation at different levels would uniformly have positive effects on spinal cord neural repair. Second, decompression surgery is one of the most effective treatments for CSM. The unresolved question is whether the simultaneous administration of pro-angiogenic drugs following decompression surgery might further improve outcomes.

## 5 Conclusion

The microvascular statuses are an effective factor in the neurological function post-CSM. The proliferation of microvessels aids neural repair.

## Data availability statement

The raw data supporting the conclusions of this article will be made available by the authors, without undue reservation.

## Ethics statement

The animal studies were approved by Animal Experiment Ethics Committee of the Affiliated Hospital of Guangdong Medical University. The studies were conducted in accordance with the local legislation and institutional requirements. Written informed consent was obtained from the owners for the participation of their animals in this study.

## Author contributions

X-xW: Data curation, Methodology, Software, Visualization, Writing – original draft. G-sL: Project administration, Writing – review & editing. K-hL: Data curation, Visualization, Writing – review & editing. X-sH: Methodology, Software, Writing – review & editing. YH: Conceptualization, Funding acquisition, Supervision, Writing – review & editing.
