# Correction: Positive effect of microvascular proliferation on functional recovery in experimental cervical spondylotic myelopathy

**DOI:** 10.3389/fnins.2026.1894728

**Published:** 2026-06-22

**Authors:** Xu-xiang Wang, Guang-sheng Li, Kang-heng Wang, Xiao-song Hu, Yong Hu

**Affiliations:** 1 Department of Minimally Invasive Spine Surgery, The Affiliated Hospital of Guangdong Medical University, Zhanjiang, China; 2 Department of Orthopaedics and Traumatology, The University of Hong Kong, Hong Kong, Hong Kong SAR, China; 3 Orthopedics Center, The University of Hong Kong-Shenzhen Hospital, Shenzhen, China

**Keywords:** neural repair, microvascular proliferation, cervical spondylotic myelopathy, chronic, recovery

The reference for Mattucci et al., 2019, in section 2.2 *Grouping and modeling*, was erroneously written as “Mattucci, S., Speidel, J., Liu, J., Ramer, M. S., Kwon, B. K., Tetzlaff, W., et al. (2019). Development of a traumatic cervical dislocation spinal cord injury model with residual compression in the rat. J. Neurosci. Methods 322, 58–70. doi: 10.1016/j.jneumeth.2019.03.010”. It should be Li et al., 2022, written as “Li, G. S., Wang, X. X., Tan, R. B., Wang, K. H., Hu, X. S., and Hu, Y. (2022). Ultrastructural destruction of neurovascular unit in experimental cervical spondylotic myelopathy. *Front. Neurosci*. 16:1031180. doi: 10.3389/fnins.2022.1031180”.

The reference for Li et al., 2022, in **Discussion** section, paragraph 2, line 6, was erroneously written as “Li, G. S., Wang, X. X., Tan, R. B., Wang, K. H., Hu, X. S., and Hu, Y. (2022). Ultrastructural destruction of neurovascular unit in experimental cervical spondylotic myelopathy. Front. Neurosci. 16:1031180. doi: 10.3389/fnins.2022.1031180”. It should be Basso et al., 1996, written as “Basso, D. M., Beattie, M. S., Bresnahan, J. C., Anderson, D. K., Faden, A. I., Gruner, J. A., et al. (1996). MASCIS evaluation of open field locomotor scores: effects of experience and teamwork on reliability. Multicenter animal spinal cord injury study. *J. Neurotrauma*. 13, 343–359. doi: 10.1089/neu.1996.13.343”.

There was a mistake in Figure 4 B2, C2 and D2 as published. There are areas in the Figure which are not present in the original images.

**Figure 4:**
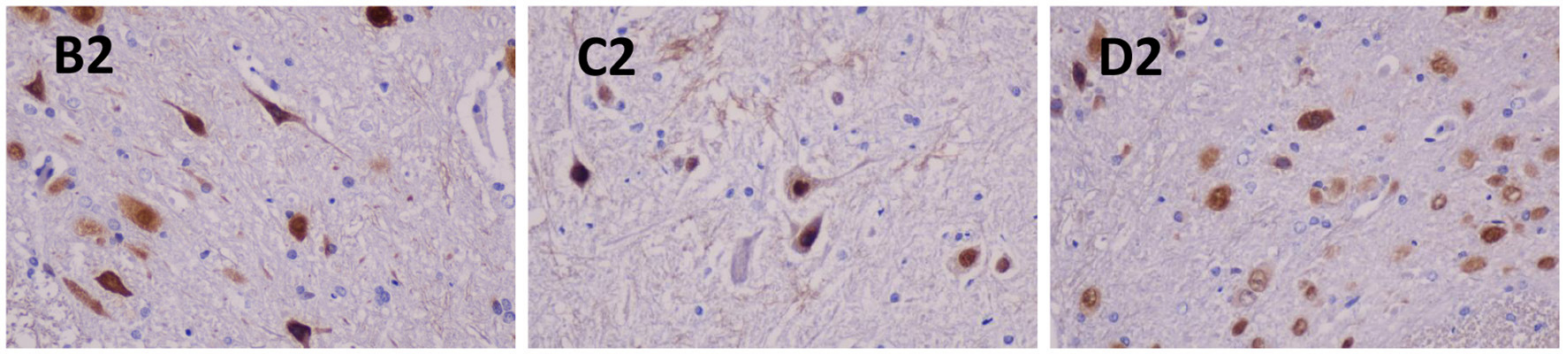
Microvascular proliferation and, thereby, NVU remodeling after DFO intervention. **(A–D, A1-D1, E, F)** Semiquantitative analysis of LEA staining and CD31 (indicated by an arrow) revealed that SU5416 intervention inhibited endothelial cell proliferation, while DFO intervention promoted it (indicated by an arrow). **(A2–D2, G)** Expression of NEUN was lower in the COM group than in the CON group and declined in the AS group. In the AG group, it increased. **(A3-D3, A4–D4, A5-D5, H, I, J)** Compared to the CON group, there was an increase in glial cells in the COM group. Glial cells decreased in response to SU5416 but increased with DFO. **(K, L)** Heatmap analysis showed that the components of NVU were closely related to motor and sensory functions, as well as micro-pathological changes in the spinal cords. Lat, Latency; Amp, Amplitude; TB, Toluidine Blue; LFB, Luxol Fast Blue; AF, Anterior funiculus; LF, Lateral funiculus; PF, Posterior funiculus; VH, ventral horn; BBB, Basso Beattie Bresnahan; NUM, number of neurons; LEA, *Lycopersicon Esculentum* Agglutinin; CD31, markers for microvessels; NEUN, markers for neurons; GFAP, markers for astrocytes; Olig2, markers for oligodendrocytes; IBA, markers for microglia; “^*^” signifies that there was statistical significance (*p* < 0.05).

The corrected Figure 4 B2, C2 and D2 appears below.

The original version of this article has been updated.

